# Supplementary material for: Repeated plague infections across six generations of Neolithic Farmers
Source: Nature. 2024 Jul 10;632(8023):114–21. doi: 10.1038/s41586-024-07651-2 (PMC11291285; doi:10.1038/s41586-024-07651-2)
Supplement: Supplementary file 1 — Supplementary Notes 1–5, Figs 1–11, legends to Tables 1–15 and references. [file 41586_2024_7651_MOESM1_ESM.docx]

# Supplementary Information

For *Repeated Plague Infections Across Six Generations of Neolithic Farmers*, Seersholm et al. 2024

The following two supplementary files accompany the paper ‘Repeated Plague Infections Across Six Generations of Neolithic Farmers’:

- Supplementary Information

- Supplementary Tables

### Supplementary Information

Supplementary Note 1 – Site Descriptions

Supplementary Note 2 - Supplementary Methods

Supplementary Note 3 - Chronological modelling

Supplementary Note 4 - Classification of the Gok2 strain

Supplementary Note 5 - An investigation of the two admixed individuals

Supplementary Figures

Supplementary References

### Supplementary Tables

Supplementary Table 1

Supplementary Table 2

Supplementary Table 3

Supplementary Table 4

Supplementary Table 5

Supplementary Table 6

Supplementary Table 7

Supplementary Table 8

Supplementary Table 9

Supplementary Table 10

Supplementary Table 11

Supplementary Table 12

Supplementary Table 13

Supplementary Table 14

Supplementary Table 15

### Supplementary Tables Legends

**Supplementary Table 1. Sequencing statistics (individual-wise).** Overview of sample information, sequencing statistics and population genetic classifications for each genetic individual.

**Supplementary Table 2. Sequencing statistics (sample-wise).** Comprehensive sample information, sequencing statistics and population genetic classifications for each sample.

**Supplementary Table 3. Sequencing statistics (library-wise).** Sequencing statistics and contamination estimates for each library.

**Supplementary Table 4. Radiocarbon dating.** Complete list of all radiocarbon dates used in this study.

**Supplementary Table 5. Strontium data.** Complete list of all strontium measures used in this study.

**Supplementary Table 6. *Yersinia pestis* mapping statistics.** Mapping statistics of reads mapping to *Yersinia pestis* in plague positive samples.

**Supplementary Table 7. Ancient human reference genomes.** Overview of sample information and sequencing statistics for all reference genomes used in this study.

**Supplementary Table 8. Overview of relevant IBD-clusters.** Sample and cluster information for all relevant IBD-clusters (clusters containing at least one sample from this study).

**Supplementary Table 9. Pairwise relatedness.** Information on pairwise relatedness for all pairs of samples sequenced for this study generated using ngsRelate.

**Supplementary Table 10. Raw data from pathogen screening results.** Raw data on pathogen hits that pass all filters, including relevant statistics used to evaluate the credibility of each hit. *coveragePRatio* is a measure of coverage evenness, quantified by normalising the observed breadth of coverage to the expected breadth of coverage given the number of mapped bases.

**Supplementary Table 11. Chronological modelling results pedigree 1, right branch.**

**Supplementary Table 12. Chronological modelling results pedigree 1, left branch.**

**Supplementary Table 13. Chronological modelling results pedigree 2, Landbogården.**

**Supplementary Table 14. Pangenome coverage statistics for all genes/regions identified.**

**Supplementary Table 15. Overview of ENA accession numbers for all publicly available fastq files from this study.** Table contains information on ENA sample accession, ENA biosample accession and ENA run accession numbers, together with sample id, library id and library strategy (WGA/capture enrichment) for all uploaded files.

## Supplementary Note 1 – Site Descriptions

### Avlebjerg, non-monumental burials; Strøby 05.06.12-8A, Zealand, Denmark Lat 55.368, Long 12.287, inv no NM A 37692-701

The Avlebjerg site is situated at the top of a hill next to a river valley. Gravel extraction in 1937 and 1939 gave rise to expedient archaeological excavations of two non-monumental burials with badly preserved human skeletal remains. During the first excavation an irregular stone cist, measuring 2.6 by 0.85 m and oriented NE-SW, was revealed. It contained the skeletal remains of two children, termed individuals I and II. They lay in opposed crouched positions facing SE. Individual I was richly supplied with burial gifts, including 15 tooth pendants (up to 8 cm long), two tubes made of bone (the largest measuring 5.7 by 1.1 cm), two ornaments made of boar tusk (length 10 and 8.5 cm, respectively) and the crumbling remains of several amber beads. Individual II was associated with two beads of amber^74,75^.

The other grave was found at a distance of 12-15 m towards the NE. It consisted of a heap of stones, covering an E-W oriented pit with the highly disintegrated bones and teeth of an approximately 8 year-old child (individual III). The skeleton was placed with the head toward the E and was surrounded by an irregular frame of stones. No burial goods were revealed in this context.

A third burial with bones of small size was destroyed by gravel diggers prior to the 1937 archaeological field work.

During a previous study individuals I and III were sampled for DNA analysis, AMS dating, strontium analysis and the measuring of dietary isotopes (δ^13^C and δ^15^N). Sufficient endogenous DNA was only found in the former (individual I, NEO961, AVL001)^23^. A physical anthropological examination determined it to represent the remains of a child, aged ca. 4½ years. Genetically it is determined female^23^. The radiocarbon date of this individual is 4510±32 uncal BP, 5305-5047 cal. BP (95.4%, UBA-40443), corresponding to the early Middle Neolithic TRB (MN I).

**Firse sten passage grave, Falköping östra 1:1, Falbygden, Sweden**Lat. 58.15302, Long. 13.56865, inventory no VGM 1M16-107079.

The grave underwent minor restoration in the 1950s and was partly excavated in 2008 by Västergötlands museum^11,76^. The chamber measured 8x2.5 m and was oriented NNE- SSW. A passage, about 8 m long, was centrally placed perpendicular on the eastern long side of the chamber and ended in two façade slabs. In front of the entrance and the facade, there was a stone-paved area. The chamber was surrounded by a mound, 30 m in diameter, and the chamber and most of the passage was covered by roof slabs.

Parts of the passage had been rebuilt into a gallery grave during the Late Neolithic, and in this section at least three individuals, two adults and one child, were documented. The skeletons seemed to be articulated and one of the skeletons lay in a supine position. Furthermore, an inhumation of an adult man was found in the surrounding mound along with bronze artefacts dated to Bronze Age period III/IV. The bones in the mound were commingled, probably due to secondary disturbance. The northern part of the chamber was also excavated and in the bottom layer of the north-eastern corner a teenage female was placed in a contracted position on her side. On the stone floor in the entrance area, TRB pottery sherds, burnt and unburnt flint blades, and unburnt human bones were recovered.

Seven ^14^C dates were previously published^11,71,77^. The burial in the mound was dated to the Scandinavian Bronze Age period III/IV. Two individuals in the rebuilt passage were dated to the Scandinavian Late Neolithic/Early Bronze Age. A human tooth in the entrance area and skeletal remains from two individuals in the chamber were dated to the Scandinavian Middle Neolithic A period. Further, Sr isotopes from four individuals have been analysed^78^.

In the present project, an M2 tooth from the Bronze Age male buried in the mound was included (F27, ID 1656, genetic indivId: FIR001). A canine tooth probably from the same individual gave an Sr isotope ratio of 0.71362, suggesting he spent his early years within the Falbygden geology^78^.

**Frälsegården passage grave, Gökhem 94:1, Falbygden**Lat 58.16393, Long 13.45733, Inv no VGM 1M16-107047

The passage grave at Frälsegården was excavated in 1999-2001 by Gothenburg University^18,19^. In spite of damage and ploughing, this constitutes the most well-documented bone material from a Scandinavian megalithic tomb.

Most of the chamber stones had been removed ca. 1900 and the site had been ploughed over since. In spite of this destruction, the construction could be documented and a large amount of bone material collected. The tomb was found to have been rectangular, approximately 9.1 x 1.8 m large, with a roughly 10 m long passage, and constructed of limestone slabs. Traces of drywalling of slate slabs were found in several places along the walls. Within the chamber a number of sections partitioning the chamber were found. The passage was divided up by thresholds in at least two places, suggesting internal doorways. The chamber had been surrounded by a mound, about 30 m in diameter. In the chamber a compact, roughly 20 cm thick bone layer with more than 10,000 fragments of bone was found. Most of the bones were measured in detail with a total station.

The presence of a number of whole or partially articulated skeletons was one of the most significant results of the excavation. These range from almost complete skeletons to partial articulations. In addition, there is a mass of disarticulated bones but also some bones that seem to have been treated differently, such as a skull group and a couple of bone packages. The number of buried individuals are estimated to be at least 51, but more likely ca 78-80^14^. Datings of the skeletons range mainly ca. 5,100-4,900 cal BP, corresponding to the late Funnel Beaker Culture, period Middle Neolithic A in the Scandinavian chronology. A large number of scientific analyses have been published, including aDNA, dietary isotopes, and Sr isotopes^11,14,18,19,23,72,79–83^. Two individuals were previously found to be infected by the plague, and Sr isotopes suggest that some 25% of the individuals were born outside the local area.

In the present project, 104 samples were included, 42 of which were analysed in Uppsala and 62 in Copenhagen (Supplementary Table 2).

**Hjelmars rör passage grave, Falköping stad 3:1, Falbygden**Lat. 58.16982, Long. 13.57805, Inventory no SHM 4032, 3510, 35151, 35152

The passage grave was partially excavated by Bror-Emil Hildebrand in 1868, and by the University of Gothenburg in collaboration with Västergötlands museum in 1994, 1995 and 1998^84^. The grave was surrounded by a mound, 24 m in diameter. The rectangular chamber oriented N-S was covered by roof slabs and measured 5.6x2.5 m. During the excavation in 1868, a large number of unburnt human bones were found accompanied by animal bones, numerous amber beads, flint blades and flint flakes. Most of this material was put back into the chamber.

In 1994 the entrance area and parts of the mound was excavated. In the mound, four secondary graves were recovered, suggested to date to the Bronze and Iron Ages. In the entrance cairn, a concentration of TRB pottery sherds, flint items, fragments from ground axes, a barbed flint arrowhead with a concave base, and a stone chisel were found, and in the upper layer a bronze tweezer was recovered^85^. In 1995-1998, the chamber was investigated and abundant human bones were found along with some pottery sherds, amber beads, two tanged blade arrowheads, and some flint flakes and flint blades^84,86,87^. During this investigation, niches were observed in the chamber. Two pottery vessels were found in the chamber, one attributed to the TRB and the other to the PWC. Three stones with cup marks were recovered in the mound.

The skeletal remains were investigated by Wilhelmson (2003)^88^ and the MNI was estimated to 26, comprising individuals of different ages and with a relatively high proportion of women. Eight individuals from the northern part of the chamber were dated to the Scandinavian Early Neolithic/Middle Neolithic A period^84,87^. One of the secondary burials in the mound was dated to the mediaeval period.

In the present project, 23 samples recovered from the chamber were included (Supplementary Table 2).

**Holma passage grave, Karleby 105:1, Falbygden**Lat. 58.19006, Long. 13.63280, Inv no VGM 1M16-107140

This passage grave was partially excavated and restored by Västergötlands museum in 2005. The grave was surrounded by a mound about 19 m in diameter. The rectangular chamber measured 6.2x2.2 m and was oriented SW-NE. A perpendicularly placed passage from the eastern long side measured 5.5x0.8m. A bronze awl was found with some cremated bones outside of the chamber and interpreted as a Bronze Age burial. Amber beads, a pottery sherd and a tanged blade flint arrowhead (type A) were also recovered during the excavations and inhumed human bones were found inside the chamber. Late Neolithic and Iron Age reuse were indicated by a barbed flint arrowhead with a concave base and an iron knife. Three individuals were previously dated, one to the LN I and two to MN A/MN B^11^.

One sample was included here, an M1 tooth from a 20-30 years old adult, dated to the late MN A (2F6293, Supplementary Table 2).

**Hunnebostrand passage grave, Tossene 210, Bohuslän**

Lat 58.4384, Long 11.3007, Inv no SHM 7532:107a+b

This is the only passage grave or dolmen in Bohuslän with preserved Neolithic bones, due to its location on a late glacial shell bank. It consists of an irregular, approximately oval chamber, ca 3m long and 1.5 m wide, with a 3.7 m long passage towards the east. The grave was investigated in 1885 by Gustavsson and restored in 1940 by Niklasson. From the 1885 excavation, human bones were recovered, estimated to represent at least 4 individuals from the chamber and 9 individuals from the passage. In 1940, around 200 pieces of human bone were recovered from the passage. No detailed osteological analysis has been made. Animal bones and one flint flake have also been recovered.

Previously, datings were published by Sjögren^15^, and Sr isotopes by Sjögren et al^72^. For the present study, three samples were analysed, one of which was dated to the early MN A (Supplementary Table 1).

**Landbogården passage grave, Gökhem 17:1, Falbygden**Lat. 58.17755, Long. 13.43455, Inventory no SHM 32201

The passage grave was excavated in 1987 by Lars Bägerfeldt. The grave consists of a small rectangular chamber, 2.7x1 m, oriented NNW-SSE, and a four m long centrally placed passage leading from the eastern chamber wall. It was constructed by limestone slabs and surrounded by a stone setting. Amber beads, flint flakes, flint blades, a flint scraper, animal teeth, and slate beads were recovered from the grave. In the mound above the passage Iron Age pottery sherds were found. In all, 857 human and 84 animal bone specimens were found, including five articulated skeletons. One individual (A) was placed on his back across the passage, and under a limestone slab further down the passage a bone package with bones from two partially articulated individuals (individual F1, subadult and F2, adult) were found^14,89^. The chamber contained 3-4 articulated individuals (individuals B-E) in contracted positions.

Osteological material from the tomb has been analysed by Ahlström^14^, light isotopes by Lidén^90^ and strontium isotopes by Sjögren et al.^72^. The minimum number of people buried in the chamber was estimated by Bägerfeldt^89^ to fourteen and by Ahlström (2009:82)^14^ to nine. The former number is preferred since Ahlström only considered postcranial bones.

Six individuals were dated in connection with the excavation at the Stockholm laboratory using radiometric technique^89,91^. The male placed across the passage was dated to the Early Iron Age. These dates all have very large standard deviations and the pretreatment used is unknown. Complementary dates and re-dates have since been performed on some of the Neolithic individuals, and are now clearly dated to the MN A^11,84,86,92^.

In this study 17 samples were analysed (Supplementary Table 2).

**Nästegårdskvarn gallery grave, Falköpings västra 1, Falbygden**

Lat 58.1633, Long 13.5219, Inv no SHM 20899

This grave, also called Nästegården, was never excavated but was subject to restoration by Einar Magnusson in 1935. The restoration revealed a rectangular chamber of limestone slabs, ca 2.7x1 m large, oriented NE-SW. It was surrounded by a low mound, ca 7 m in diameter, with a circle of rounded kerb stones. The architecture permits a classification as both a dolmen and a gallery grave, since the definitions of these grave types overlap. But since the datings are all Late Neolithic, we class it as a gallery grave.

The chamber content had been subject to recent disturbance, but human bones were recovered outside the chamber, on the mound surface, together with animal bones, some flint objects, four bone needles and ca. 10 small pottery sherds.

Six samples were included in the present project. These were all dated to the Late Neolithic (Supplementary Table 2).

**Rössberga passage grave, Valtorp 2:1, Falbygden**Lat. 58.22951, Long. 13.60576, Inventory no: SHM 27911

This is the only passage grave in Falbygden where the surrounding cairn and the whole chamber and passage was excavated. The investigation was conducted by Cullberg in 1962^84,93^. The grave was still covered by roof slabs and the 9x2 m large rectangular chamber was oriented N-S with an 8 m long passage placed perpendicular to the eastern chamber wall, continuing in a facade. The chamber was divided into small compartments by more than 30 limestone slabs.

In front of the passage, in the entrance cairn, approximately 800 TRB pottery sherds from at least 65 vessels, burnt and unburnt flint flakes and other flints, a tanged blade flint arrowhead, several pieces of polished stone and flint axes, a recut Late Neolithic flint dagger, a few BAC pottery sherds, and cremated bones were documented^93^. In the chamber human bones, tooth pendants, a bone needle, amber beads, some flint items, and a piece of an ornamented bone ring of BAC type were recovered^93^.

According to Cullberg, none of the human bones were articulated. The bones were assigned to units (Be) representing the suggested compartments of the chamber. Human bones were also found in the passage along with some flints and amber beads. The bone material was investigated by Ahlström^13,14^ and the MNI was estimated to 131 (men, women and children). This is the largest number of individuals recorded from any Scandinavian megalithic tomb.

Several series of datings and isotope measurements were previously published^11,72,78,84,92,94,95^. Genomic and mtDNA analyses were published by Malmström et al.^96^ and Blank et al.^78^.

25 samples were included in the present study. These were analysed in Uppsala (Supplementary Table 2).

## Supplementary Note 2 - Supplementary Methods

### Chromosome Y haplogroups assignments

Chromosome Y haplogroups were assigned using an in-house pipeline developed for this study. For each individual identified as male with a chromosome Y coverage over 0.002X, bam files were subset to reads mapping to the Y chromosome (samtools view) followed by SNP calling with bcftools mpileup and bcftools call. The resulting VCF file were then subset to ISOGG snps (https://docs.google.com/spreadsheets/d/1UY26FvLE3UmEmYFiXgOy0uezJi_wOut-V5TD0a_6-bE/edit#gid=1934392066), and for each derived allele the corresponding ISOGG subgroup name (e.g. I2a1a1b1a1a3~) was saved. Followingly, for each sample, ISOGG subgroups were counted, and grouped into lists of subgroups that are not mutually exclusive. For example, for haplogroup I2a1a2a1a1a SNPs supporting the following ISOGG subgroups counts towards the total count of this haplogroups: I, I2, I2a, I2a1a, I2a1a2, I2a1a2a, I2a1a2a1a, I2a1a2a1a1, I2a1a2a1a1a, I2a1a2~, I2~. Lastly, chromosome Y haplogroups were simply assigned to individuals by selecting the haplogroup supported by the highest number of SNPs.

###

### Pedigree reconstruction

#### Reconstruction of 1st degree pedigrees at Frälsegården, pedigree 1

To reconstruct pedigrees from information of pairwise relatedness, we started out by plotting a network of only 1st degree relations. Using this data, we were able to reconstruct unambiguous pedigrees for most clusters of 1st degree relatives (see Supplementary Figure 3). In two cases, however (subpedigrees d and g), it was not possible to establish who was the parent and who was the offspring in parent-offspring relations between males. To solve these ambiguities, we combined relatedness data with both radiocarbon dates, burial location and connections to 2nd and 3rd degree relatives for each individual:

**Subfamily d.** FRA011 and FRA012 have a parent-offspring (PO) relationship, since both are males, it is not possible to use mitochondrial haplogroups to establish who is the parent and who is the child. Similarly, radiocarbon dates are not informative as they have overlapping uncertainties (FRA011: 4439 ± 30 uncal. BP, FRA012: 4455 ± 15). However, FRA011 was classified as an adult while FRA012 was classified as juvenile, suggesting that FRA011 is the father of FRA012.

**Subfamily g.** FRA021 and FRA024 both have a PO relationship with FRA022 but are 2nd degree relatives to each other. Therefore, FRA021 and FRA024 can either be half-brothers or grandparent-grandchild. To establish whether FRA021 and FRA024 are half siblings, we can use the fact that FRA022 is a third degree relative to the mother (FRA023) of his other children (FRA025 and FRA026). If FRA021 and FRA024 were half-brothers, they would be equally related to FRA023. This is not the case. FRA024 is a 3-4th degree relative to FRA023, while FRA021 and FRA023 appear to be unrelated. This is consistent with a pedigree where FRA021 is the father of FRA022 and grandfather of FRA024, while FRA023 and FRA022 are related through the mother of FRA022. This is supported by 1) The fact that FRA021 was buried under a limestone slab right beneath FRA022 and 2) radiocarbon dates suggesting that FRA024 is younger than FRA021 and FRA022 who have similar ages (FRA21: 4420±20 and 4445±15, FRA022: 4425 ± 15, FRA024: 4385 ± 15).

#### Reconstruction of full pedigrees at Frälsegården, pedigree 1 (left subfamily)

**Subfamilies a and b.** FRA002 and FRA004 are second degree relatives with similar radiocarbon dates (FRA002: 4455 ± 20 and 4485 ± 15, FRA004: 4460± 20). They could be either uncle/nephew, grandparent/grandchild, or half-siblings. Both are 2nd degree relatives to FRA011 and third-degree relatives to the progenitor of the right subfamily FRA021. Under the assumption that FRA011 and FRA021 are placed higher in the pedigree than they are, they must be half siblings. If they had an avuncular relationship there would be one relatedness degree difference to FRA011 and FRA021 between the two (e.g. 2nd and 3rd degree), if they were grandparent/grandchild there would be two degrees of relatedness difference to FRA011 and FRA021.

**Subfamilies a,b and c.** FRA004 (male) and FRA007 (female) are both second degree relatives of siblings FRA009 and FRA010. As they are equally related to both siblings, they must be first degree relatives of either the father or mother of the siblings. Both are unrelated to the mother (FRA008), hence they are first degree relatives of the father (unsampled). There are three options, grandparent, aunt/uncle or half sibling. Except for the scenario where both FRA004 and FRA007 are grandparents, they would be either first or second degree relatives to each other. As FRA004 and FRA007 are unrelated, they must be paternal grandparents to FRA009 and FRA010.

**Subfamilies a,b,c and d.** The halfsiblings FRA002 and FRA004 are second degree relatives to FRA011. The relationship between FRA002/FRA004 and FRA011 can be a) half siblings, b) grandparent/grandchildren or c) uncle/nephews.

To solve this pedigree, we use the fact that FRA002, FRA004 and FRA011 are all related to the progenitor of the right subfamily (FRA021): While FRA002 and FRA004 are third degree relatives to FRA021, FRA011 is a second degree relative to FRA021. Hence, their relationship must be uncle/nephews. If they were all half-siblings, they should be equally related to FRA021, and if they were grandparent/grandchildren the grandchildren should be equally related to FRA021 while the grandparent should be 2 steps of relatedness differently related to FRA021 than the grandchildren.

**Subfamilies a,b,c,d and e.** FRA014 and FRA015 are 2nd degree relatives of FRA011 and third degree relatives of the son of FRA011, FRA012. Because there are no other close relatives of FRA014 and FRA015, it is not possible to establish how they are related to FRA011.

Reconstruction of full pedigrees at Frälsegården, pedigree 1 (right subfamily)

**Subfamilies f and g.** The two brothers FRA039 and FRA040 are 2nd degree relatives to the father and son FRA021 and FRA022. The only scenario where an individual is a 2nd degree relative to both individuals in a parent-offspring pair, is when that individual is grand-child to the parent and nephew to the offspring. Hence FRA039 and FRA040 must be nephews to FRA022 and grandsons of FRA021. As FRA039 and FRA040 do not share mitochondrial haplogroups with their uncle FRA022, they must be related through the father of FRA039 and FRA040.

**Subfamilies h and j.** FRA027 and FRA028 are both 2nd degree relatives to the group of siblings FRA032, FRA033, FRA034 and FRA035. As FRA027 and FRA028 are unrelated to the mother of these four siblings (FRA029), they must be first degree relatives to the father of the siblings. As FRA027 and FRA028 are unrelated, they must be parents to the father of the group of siblings.

**Subfamilies h,j and i.** Siblings FRA030 and FRA031 and siblings FRA032, FRA033, FRA034, FRA035 are 2nd degree relatives of each other. Since these two groups of siblings are equally related, they must be related through their parents. Furthermore, as FRA030 and FRA031 are unrelated to the mother of FRA032, FRA033, FRA034 and FRA035, the two groups of siblings must be related through the father of FRA032, FRA033, FRA034 and FRA035. Hence, FRA030 and FRA031 must be first degree relatives to the father of FRA032, FRA033, FRA034 and FRA035. They cannot be his parents as his parents are FRA027 and FRA028, but they could be his siblings or his children. If they were his siblings, their mother and father should be FRA027 and FRA028 which is not the case. Accordingly, they must be his children, and thus half-siblings to FRA032, FRA033, FRA034 and FRA035.

**Subfamilies f,g and h,j,i.** These two subfamilies are connected through FRA027, who is a second degree relative to the father and son FRA021 and FRA022. The only scenario where an individual is a 2nd degree relative to both individuals in a parent-offspring pair, is when that individual is grand-child to the parent and nephew/niece to the offspring. Hence FRA027 must be the nephew of FRA022 and grandson of FRA021. As FRA027 do not share mitochondrial haplogroup with his uncle FRA022, they must be paternally related.

**Subfamily f,g,h,j,i and individual Gok4.** Gok4 is a second degree relative to FRA040 and a third degree relative to the brother of FRA040 (FRA039). The only possible scenario where an individual is a second degree relative to one sibling but only a third degree relative to the other sibling, is if that individual is the grand-child of the first sibling. Hence, Gok4 must be the grandson of FRA040. It is not possible to determine whether they are related through the mother or the father of Gok4.

**Subfamily f,g,h,j,i and individual FRA037.** FRA037 is a second degree relative to both FRA027 and FRA028 and a third degree relative to the brothers of FRA028 (HJE003 and HJE012). The only possible scenario where an individual is a second degree relative to one sibling but only a third degree relative to the other siblings, is if that individual is the grand-child of the first sibling. Hence, FRA037 must be the grandson of FRA027 and FRA028. As FRA037 does not have the same mitochondrial haplogroup as FRA028, FRA028 and FRA027 must be his paternal grandparents.

Reconstruction of pedigree 2 (Landbogården)

**LAN001, LAN002, and LAN003.** LAN001 and LAN002 both have a P-O relationship to LAN003, and are not related to each. Accordingly, LAN001 and LAN002 must be parents of LAN003.

**LAN001/LAN002/LAN003 and LAN004/LAN005.** LAN004 and LAN005 are siblings, and both are second degree relatives to LAN001, LAN002 and LAN003. The only scenario where an individual is a 2nd degree relative to both individuals in a parent-offspring pair, is when that individual is grand-child to the parent and nephew/niece to the offspring. Hence, LAN004 and LAN005 must be grandchildren of LAN001 and LAN002. As they do not share a mitochondrial haplogroup with these grandparents, LAN001 and LAN002 must be their paternal grandparents.

**LAN001/LAN002/LAN003/LAN004/LAN005 and LAN006.** LAN006 is a second degree relative to siblings LAN004 and LAN005, and a third degree relative to LAN001, LAN002, and LAN003. Results from KIN specifies the relationship as avuncular. Hence LAN006 could be either maternal uncle, paternal uncle or nephew to LAN004/LAN005. Paternal uncle can be ruled out, as LAN006 is not the son of the grandparents to LAN004/LAN005. Maternal uncle can also be ruled out as LAN006 does not share mitochondrial haplogroups with LAN004/LAN005. Hence, LAN006 must be a nephew to LAN004/LAN005, and since LAN006 has a different haplogroup than LAN004/LAN005, his father must be the brother of LAN004/LAN005.

Reconstruction of pedigree 3 (Rössberga)

**ROS039, ROS018 and ROS021.** ROS039 and ROS018 are siblings, and they are both second degree relatives to ROS021. As they all share mitochondrial haplogroups, they must be related through the mother of ROS039 and ROS018.

Reconstruction of pedigree 4 (Rössberga)

**ROS030, ROS024 and ROS014.** ROS024 and ROS014 have a P-O relationship. As ROS024 is a male and ROS014 is a female, and as they do not share mitochondrial haplogroups, ROS024 must be the father of ROS014. Furthermore, ROS030 is an unknown second-degree relative to ROS024 and an unknown third degree relative to ROS014.

### *Yersinia pestis* MQ0 mask

In order to account for highly repetitive regions in the *Yersinia pestis* genome, we masked out positions with high proportions of mapping quality zero reads. To generate this mask, we merged data from the highest coverage shotgun samples (individual FRA005, FRA013, FRA021, and Gok2) and removed all duplicate reads by treating all data as coming from the same library. Next, we calculated coverage at each position using mosdepth with mapping quality filters of 0 and 1, respectively. Using this data, we calculated the proportion of reads with mapping quality zero at each position. After some initial testing, we decided to filter out all regions where the proportion of mapping quality zero reads is over 2 times higher than reads with mapping quality 1 or above. Lastly, we also filtered out the region from position 3,000 to position 4,200 on the PCP1 plasmid, as this region can be problematic because of high similarity to expression vectors^34^.

### A co-infection with *Y. pestis* and *Y. enterocolitica*

Our pathogen screening pipeline identified the presence of *Y. enterocolitica* at a coverage of 2.26X for individual FRA013 (Supplementary Table 10). Surprisingly, upon a closer look at the plague hits that did not pass screening filters, we found that this sample also had 3.24X coverage of *Y. pesti*s. Our pathogen screening pipeline is designed to identify a single best hit from each genus. Accordingly, only the identification of *Y. enterocolitica* was retained, because it had the highest number of unique k-mers. This finding highlights one of the disadvantages of this screening approach, as it is not able to detect co-infections with species from the same genus. To filter out *Y. enterocolitica* reads from the *Y. pestis* bam file for FRA013, we realigned all putative plague reads to *Y. enterocolitica*. All reads with a lower number of mismatches (NM tag in bam file) in the *Y. enterocolitica* alignment than in the *Y. pestis* alignment were removed from the *Y. pestis* alignment.

### Simulated data

#### Simulated first generation individuals

In order to simulate first generation offspring of admixture between Pitted Ware Individuals and Neolithic farmers, we simulated reads from pairs of PWC/Neolithic individuals using NGSNGS^97^ as outlined below:

| **sampleId** | **sourceId A (Neolithic)** | **sourceId B (PWC)** |
| --- | --- | --- |
| sim 1 | FRA107 (this study) | ajv28 (Coutinho et al. 2020) |
| sim 2 | FRA105 (this study) | ajv36 (Coutinho et al. 2020) |
| sim 3 | FRA104 (this study) | Ajv54 (Malmström et al. 2019) |
| sim 4 | LAN007 (this study) | Hem001 (Coutinho et al. 2020) |
| sim 5 | HUN001 (this study) | Ajv58 (Skoglund et al. 2014) |

For each source, we generated a haploid consensus sequence by applying variants from the first allele of each imputed genotype to the hg37 genome (*bcftools consensus --sample [sample] -H 1 [imputedVcf] [hg37]*). We then used NGSNGS to simulate fastq read files from each of these haploid consensus sequences. To simulate the characteristics of the suspected F1-individual FRA108, we simulated reads of the same size distribution and the same coverage as FRA108 (half of the coverage from each source). Followingly, we processed the resulting fastq files using the same pipeline of mapping, filtering and imputation as the main data set. Lastly, we calculated basic mapping statistics, and plotted the resulting imputed genomes on a PCA to confirm that the simulations had worked as expected.

#### Simulated plague data

To estimate the effects of low coverage and high levels of ancient DNA damage, we simulated plague reads with the same length distributions and damage profiles as FRA005, FRA013, FRA020, FRA102 and gok002. We used NGSNGS^97^ to simulate the data with the *Yersinia pestis* reference (GCF_000009065) as the source genome for the simulations. For each of the five individuals we carried out 100 replicate simulations using a different seed each time. We then mapped the simulated reads back to the *Y. pestis* reference genome following the same pipeline as the main data set. Next, we called genotypes on the simulated data and the reference data following the same GATK based approach as for the main data set. Lastly, we counted the number of alternative alleles called for each of the simulated genomes.

### DATES analysis of admixture timing

To analyse admixture times using DATES^24^ we first subsampled plink files of the full imputed data panel to relevant target or source individuals and to 2,086,279 ﻿transversion-only SNPs with a minor allele frequency over 0.1%. We then converted the resulting plink files to eigenstrat format using *convertf*^98,99^ and ran DATES with recommended parameters. Lastly, to estimate the absolute date of admixture, we used a generation time of 25 years and the median radiocarbon date for sample-wise estimates, or the average date for the group for group-wise estimates.

### Local ancestry estimation with RFMix

Local ancestry inference was carried out with RFMix^100^ using pitted ware individuals and Neolithic individuals from this study as sources. The analyses was run on the two admixed individuals FRA108 and ROS027, together with an individual with no evidence of recent admixture (ROS016) as a control. Furthermore, the same analysis was also run on the five simulated F1-individuals described above. In order to better visualise local ancestry patterns only chromosomes one to five are shown in Extended Data Fig. 1, but all chromosomes show similar patterns of local ancestry.

### Molecular dating analyses

#### BEAST dating

Molecular dating with BEAST^101^ was carried out following Andrades-Valtueña et al. (2022)^8^ on LNBA- and preLNBA plague strains. Briefly, we generated a SNP alignment subset to LNBA- and preLNBA strains from the full alignment fasta. We used BEAUti (v.2.7.5) to set up the molecular dating parameters: As starting tip dates we used the mean calibrated radiocarbon date for all samples (Supplementary Table 4), and to account for the uncertainty of each date we included a ‘Sampled ancestor MRCA Prior’ for each sample with a uniform distribution from the lower to the upper bound of the calibrated date (2σ). We used a Coalescent Bayesian Skyline demographic model, and assumed an Optimised Relaxed Clock and the GTR substitution model with four gamma categories and empirical frequencies. We ran BEAST (v.2.7.5) with a pre burn-in of 1,000,000 states until all effective sample sizes had reached a minimum of 200. The run converged after 1,293,776,000 states.

#### BactDating analysis

As an alternative to BEAST we also tested molecular dating with BactDating^102^. As input for this analysis we generated a raxml tree of LNBA- and preLNBA strains following the same approach as the full plague phylogeny (see Methods). We used the lower and upper bound of the calibrated radiocarbon dates (2σ) as tip dates. Before running the main analysis we checked the temporal signal in the data by running a root to tip analysis, which showed a strong correlation between sample age and distance to the root (R^2^:0.98, Supplementary Figure 11). Next, BactDating (v.1.1.1) was run for 1,000,000 iterations using the ‘relaxedgamma’ model. After completion of the run, we confirmed that the analysis had converged by checking that the effective samples sizes of alpha and mu were both over 200.

## Supplementary Note 3 - Chronological modelling

###

The right side branch in Figure 3 was modelled to span a period of less than 150 years (68.3%, 1σ) and less than 170 years (2σ), with a median of 113 years and a probability peak at around 120 years. The start of this branch was modelled to 5001-4,975 cal BP (1σ) and 5044-4964 (2σ), while the end was estimated to 4,900-4,851 cal BP (1σ) and 4963-4840 cal BP (2σ).

The first three generations of the left side branch were modelled to cover less than 107 years (1σ) and less than 224 years (2σ), with a median of 70 years. The fourth generation was only represented by one outlier date. The start was modelled to 5,053-4,992 (1σ) and 5118-4980 cal BP (2σ), and the end to 4,971-4,918 (1σ) and 5028-4853 cal BP (2σ). This suggests that the left side branch probably started somewhat earlier than the right branch, after which they overlap.

The Landbogården pedigree was modelled to span <87 years (1σ) and <193 years (2σ) centred around 4,950 cal BP, overlapping with the pedigree at Frälsegården.

Further details are found in Supplementary Figure 7-Supplementary Figure 9 and Supplementary Table 13.

### Oxcal code

#### Frälsegården, right branch

Plot()

{

Sequence("Frälsegården right branch")

{

Boundary("Start");

Phase("Generation 1")

{

R_Combine("FRA021")

{

R_Date("FRA021 petrous",4445,15);

R_Date("FRA021 PM3",4420,20; Offset 20 5;);

};

};

Date("Generation 1"){color="green";};

Phase("Generation 2")

{

R_Date("FRA023 M1",4480,15; Offset 20 5;){Outlier();};

R_Date("FRA022 petrous",4425,15);

};

Date("Generation 2"){color="green";};

Phase("Generation 3")

{

R_Date("HJE012 petrous",4470,20){Outlier();};

R_Date("FRA040 mand",4415,20);

R_Date("FRA026 mand",4400,20);

R_Date("FRA028 M1",4400,15; Offset 20 5;);

R_Date("FRA039 petrous",4385,15);

R_Date("FRA025 PM4",4395,15; Offset 20 5;);

R_Date("HJE003 tooth",4395,20; Offset 20 5;);

R_Date("FRA024 M1",4385,15; Offset 20 5;);

R_Date("FRA027 mand",4365,15);

R_Date("FRA020 M1",4360,20; Offset 20 5;);

};

Date("Generation 3"){color="green";};

Phase("Generation 4")

{

R_Date("FRA041 mand",4400,20);

R_Date("FRA029 M1",4400,15; Offset 20 5;);

R_Combine("FRA042")

{

R_Date("FRA042 M1",4404,21; Offset 20 5;);

R_Date("FRA042 PM4",4365,20; Offset 20 5;);

};

};

Date("Generation 4"){color="green";};

Phase("Generation 5")

{

R_Date("FRA035 mand",4395,20);

R_Date("FRA031 petrous",4395,20);

R_Date("FRA034 petrous",4390,20);

R_Date("FRA032 petrous",4385,20);

R_Date("FRA030 M1",4390,15; Offset 20 5;);

R_Date("FRA033 petrous",4340,15);

R_Date("FRA044 mand",4315,40);

};

Date("Generation 5"){color="green";};

Boundary("End");

};

Span("Frälsegården right branch");

};

Frälsegården, left branch

Plot()

{

Sequence("Frälsegården left branch")

{

Boundary("Start");

Phase("Generation 1")

{

R_Date("FRA011 mand",4439,30);

};

Date("Generation 1"){color="green";};

Phase("Generation 2")

{

R_Date("FRA002a M1",4485,15; Offset 20 5;){Outlier();};

R_Date("FRA004 PM4",4460,15; Offset 20 5;);

R_Date("FRA007 cranium",4440,30);

R_Date("FRA002 M1",4455,20; Offset 20 5;);

R_Date("FRA012 M1",4455,15; Offset 20 5;);

R_Date("FRA001 mand",4430,20);

};

Date("Generation 2"){color="green";};

Phase("Generation 3")

{

R_Date("FRA006 mand",4405,15);

R_Date("FRA005 M1",4400,20; Offset 20 5;);

R_Date("FRA003 mand",4375,20);

R_Date("FRA008 femur",4368,27);

};

Date("Generation 3"){color="green";};

Phase("Generation 4")

{

R_Date("FRA009 petrous",4445,15){Outlier();};

};

Boundary("End");

};

Span("Frälsegården left branch");

};

Landbogården

Plot()

{

Sequence("Landbogården")

{

Boundary("Start");

Phase("Generation 1")

{

R_Date("LAN002 petrous",4405,20);

R_Combine("LAN001")

{

R_Date("LAN001 M2",4405,20; Offset 20 5;);

R_Date("LAN001 humerus",4368,28);

};

};

Date("Generation 1"){color="green";};

Phase("Generation 2")

{

R_Date("LAN003 M1",4405,15; Offset 20 5;);

};

Date("Generation 2"){color="green";};

Phase("Generation 3")

{

R_Date("LAN004 petrous",4410,15);

R_Date("LAN005 petrous",4390,15);

};

Date("Generation 3"){color="green";};

Phase("Generation 4")

{

R_Date("LAN006 petrous",4380,15);

};

Date("Generation 4"){color="green";};

Boundary("End");

};

Span("Landbogården");

};

##

## Supplementary Note 4 - Classification of the Gok2 strain

It is challenging to assign the previously published Gok2 genome^4^ to a specific plague strain from this study (strain A, B, or C), because of its relatively low depth of coverage (1.82X on average) in combination with its high rates of C to T and G to A misincorporations at the 5’ and 3’ ends, respectively. As indicated in Supplementary Figure 10, the Gok2 genome shares the highest similarity with genomes of strains C and B. But the number of different SNPs between Gok2, FRA013, FRA005 and FRA020 does not readily allow for a classification of Gok2, and the distances between these genomes are internally inconsistent because of missing data. E.g. based on the relatively limited number of SNPs called for Gok2, this genome is 100% identical to FRA020 (strain B) and FRA013 (strain C) but differs from FRA005 (strain C) on three positions (see Extended Data Fig. 8c). As depicted in the figure, these SNPs share multiple similarities that might question their authenticity: 1) All three SNPs are C>T substitutions also caused by ancient DNA damage, 2) All three SNPs are covered only by three reads, which is the minimum threshold required to call a SNP in our pipeline, 3) of a total of nine reads covering the SNPs in question, 5 reads have the alternate allele within 5bp of the read end. 4) Even though none of these SNPs were called in FRA020 and FRA013 both samples have reads supporting the reference allele. Based on these observations, we hypothesised that these three SNPs were incorrect base calls arising from C to T misincorporations.

In order to test this hypothesis, we simulated data with similar sequencing profiles as the samples FRA102, FRA020, FRA013, FRA005 and Gok2. For each sample, we simulated 100 replicates of *Yersinia pestis* fastq files with the same damage patterns, read length distributions and mean depth of coverage as the original sample, using the Yersinia pestis reference genome (GCF_000009065.1) as template (see Supplementary Note 2 - Supplementary Methods). We then processed resulting fastq files following our pipeline for mapping and basecalling of plague data. As depicted in Extended Data Fig. 8b, we found that between zero and five incorrectly called SNPs (mean: 1.06) were called for Gok2, while no incorrect SNPs were called for the four other genomes (FRA102, FRA020, FRA013, and FRA005). Based on this analysis, we conclude that the three SNPs unique to Gok2 are most likely incorrectly called variants due to a combination of high damage and low coverage.

Having established that the SNPs unique to Gok2 are less reliable, we next turned to the SNPs unique to FRA020 (strain B) to classify Gok2 as either strain B or strain C. Of the three SNPs distinguishing strains B and C, only one SNP (position 726,406 on the main chromosome, NC_003143.1) is covered by reads from Gok2. The two reads from Gok2 covering position 726,406 both support the allele from strain C (Extended Data Fig. 8d). Although this position is covered by only two reads from Gok2 at this position, we note that the SNP is an A->G substitution, and that the variant is located in the middle of both reads, suggesting that this pattern is unlikely to have arisen from ancient DNA damage. Accordingly, we tentatively assign Gok2 to strain C.

## Supplementary Note 5 - An investigation of the two admixed individuals

We identify significant proportions of hunter-gatherer DNA in the two individuals FRA108 and ROS027, both of which appear to be shifted towards individuals of the Pitted Ware Culture on our PCA plot (Figure 2). As the Pitted Ware Culture coexisted with Neolithic Farmers in Sweden for at least 600 years^103^, and given the high proportions of hunter-gatherer DNA in these individuals, we hypothesised that a relatively recent admixture event must have occurred. In order to investigate this theory, we ran DATES^24^ on the two admixed individuals and one Neolithic individual with no evidence of recent admixture (ROS016) as a control. As source populations we used individuals of the Pitted Ware culture and Neolithic individuals from this study (see Supplementary Note 2 - Supplementary Methods). Using this approach we found evidence for very recent admixture in ROS027 (2.6 ± 2.9 generations ago; Extended Data Fig. 1a), while for FRA108, we did not get meaningful results (−11.5 ± 55 generations ago; Extended Data Fig. 1a). In the case of an F1-individual, DATES would fail since there is no crossover between the two ancestries in a first generation individual. Hence, the negative result from DATES for FRA108 may suggest that this individual is in fact a first-generation Neolithic/Hunter-gatherer offspring. Similarly, with the results from DATES we can rule out that ROS027 is a first generation offspring, and given the low estimate of time of admixture (2.6 ± 2.9 generations ago), it is most likely that ROS027 represents an F2- or F3 individual.

In order to further investigate how DATES behaves in the case of an F1-individual, we simulated DNA reads of five F1-individuals with the same coverage as FRA108 (see Supplementary Note 2 – Supplementary Methods). As depicted in Extended Data Fig. 1a, DATES produced highly different results for each of the five simulated F1-individuals, and thus this test did not bring us closer to characterise the ancestry of FRA108.

Next, we decided to test RFMix^100^ to paint local ancestry across the genomes of the two admixed individuals using Pitted-Ware hunter-gatherers and Neolithic Farmers as sources (Extended Data Fig. 1b-d). We found that FRA108 had 47.5% hunter-gatherer DNA and 52.5% Neolithic DNA, while ROS027 had 34.1% hunter-gatherer DNA and 65.9% Neolithic DNA. In a perfectly phased F1-individual, each position across the genome should have one allele each of the two ancestries. This is not what we observe for the suspected F1-individual FRA108, instead we only found this pattern in 50.4% of the genome (Extended Data Fig. 1d). However, the genome of FRA108 is only at 1.3X coverage, and is not perfectly phased. Hence, to test the effects of the imputation in combination with the relatively low coverage for these types of analyses, we ran RFMix on the five simulated F1-individuals described above. These five simulated genomes behaved similarly as FRA108, both in terms of ancestry proportions and the proportion of ‘ancestry-wise heterozygous’ positions. Hence, based on these observations, we tentatively conclude that FRA108 is the first-generation offspring of parents of the Pitted Ware Culture and of the Funnel Beaker culture.

## Supplementary Figures


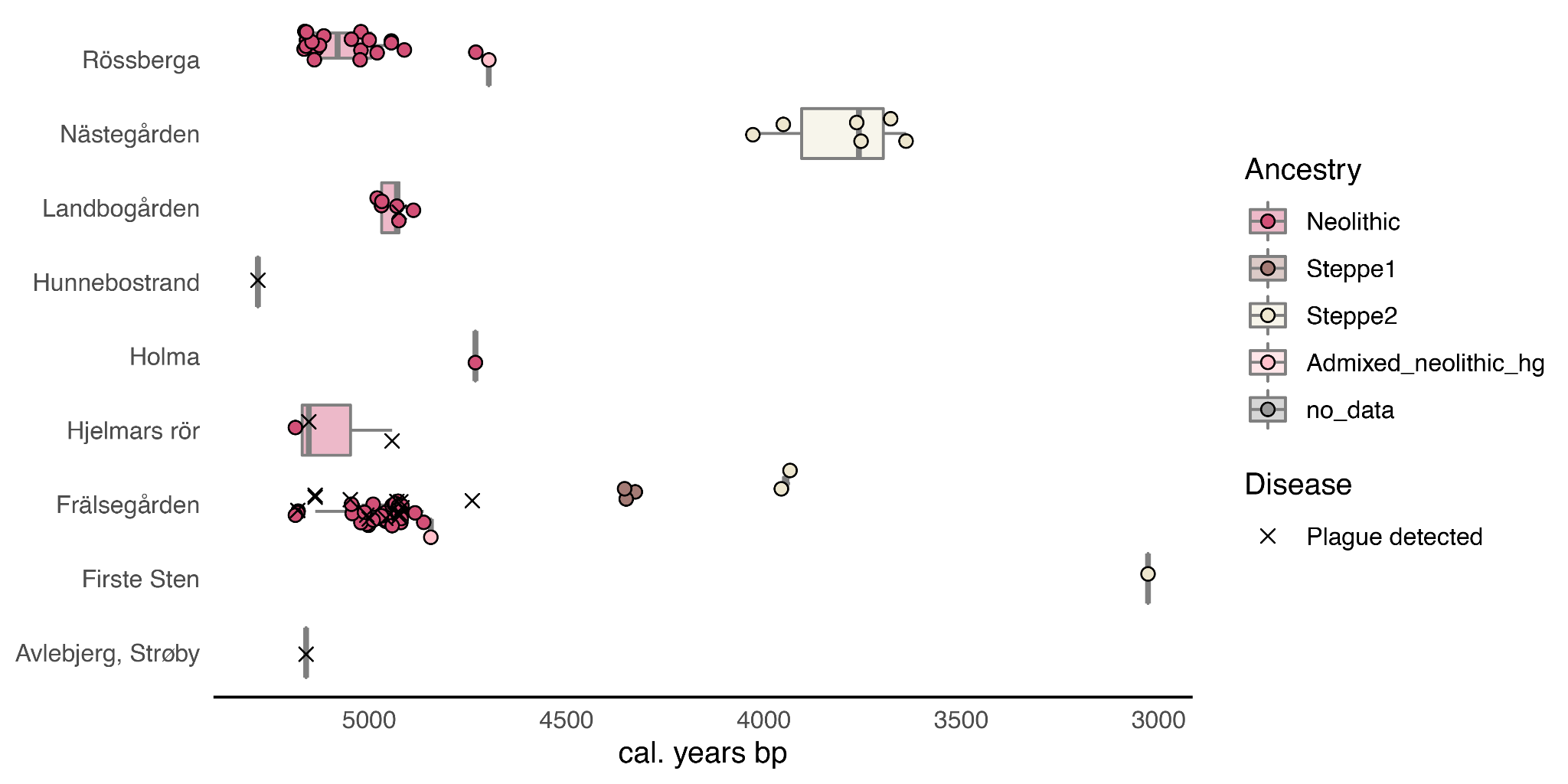


**Supplementary Figure 1. Radiocarbon dating results stratified by site and ancestry.** Each combination of site and ancestry was visualised with boxplots, with each measurement plotted on top as either coloured dots of black crosses, for individuals where no plague was detected and where plague was detected, respectively. Centre line: median. Box limits: upper and lower quartiles.


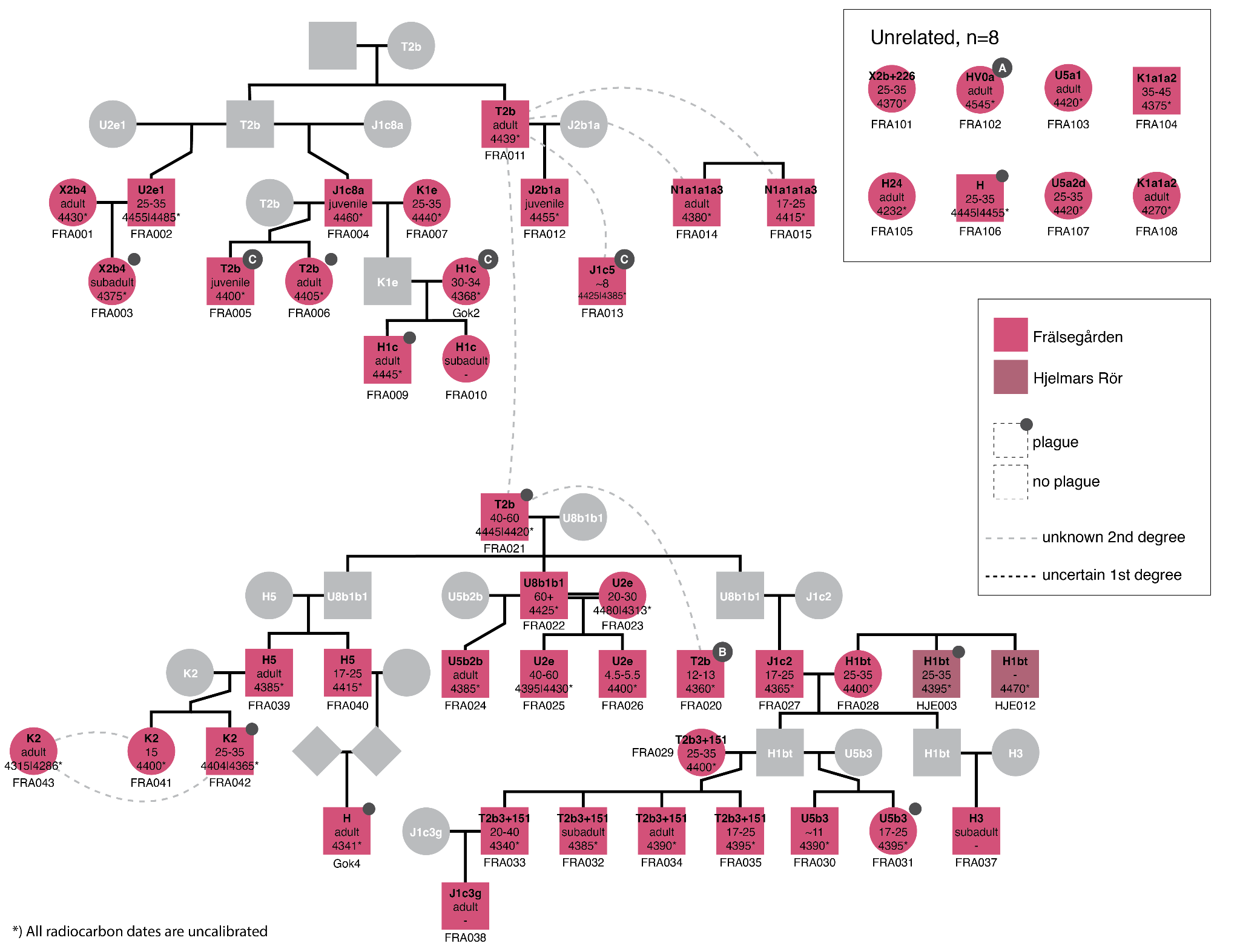


**Supplementary Figure 2. Detailed view of pedigree 1.** Squares and circles represent males and females, respectively, and information on mitochondrial haplogroup, osteological age estimate, and uncalibrated radiocarbon date for each individual is indicated inside each shape. Pink and brown colours indicate the sites Frälsegården, and Hjelmars Rör, respectively, while grey colour represents unsampled individuals. Solid black lines indicate first degree relations. Dashed grey lines signify unknown 2^nd^ degree relationships and double black lines indicate mating between related individuals.


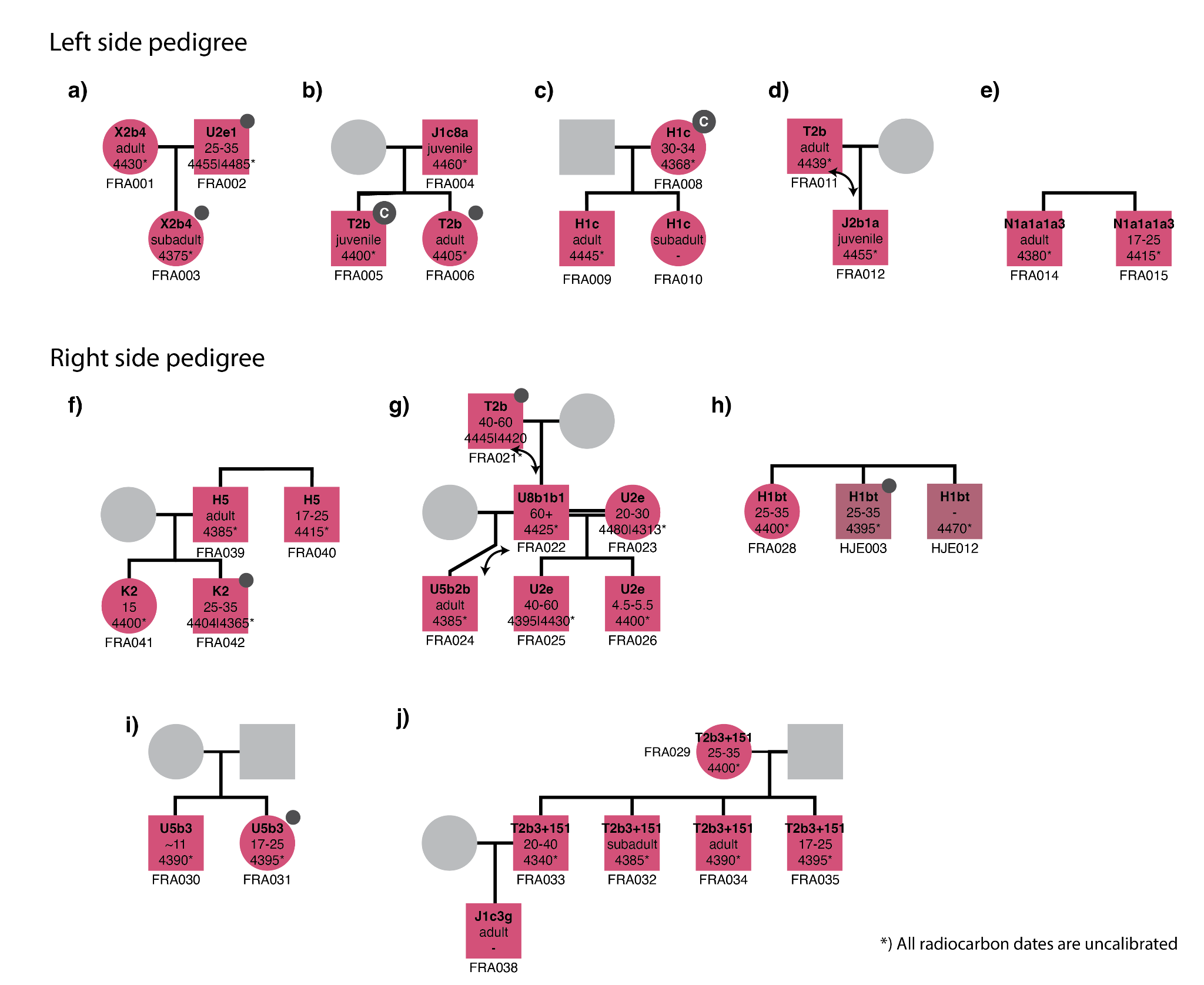


**Supplementary Figure 3. Pedigrees of only 1st degree relatives.** Arrows indicate uncertain relations between parent-offspring pairs.

**
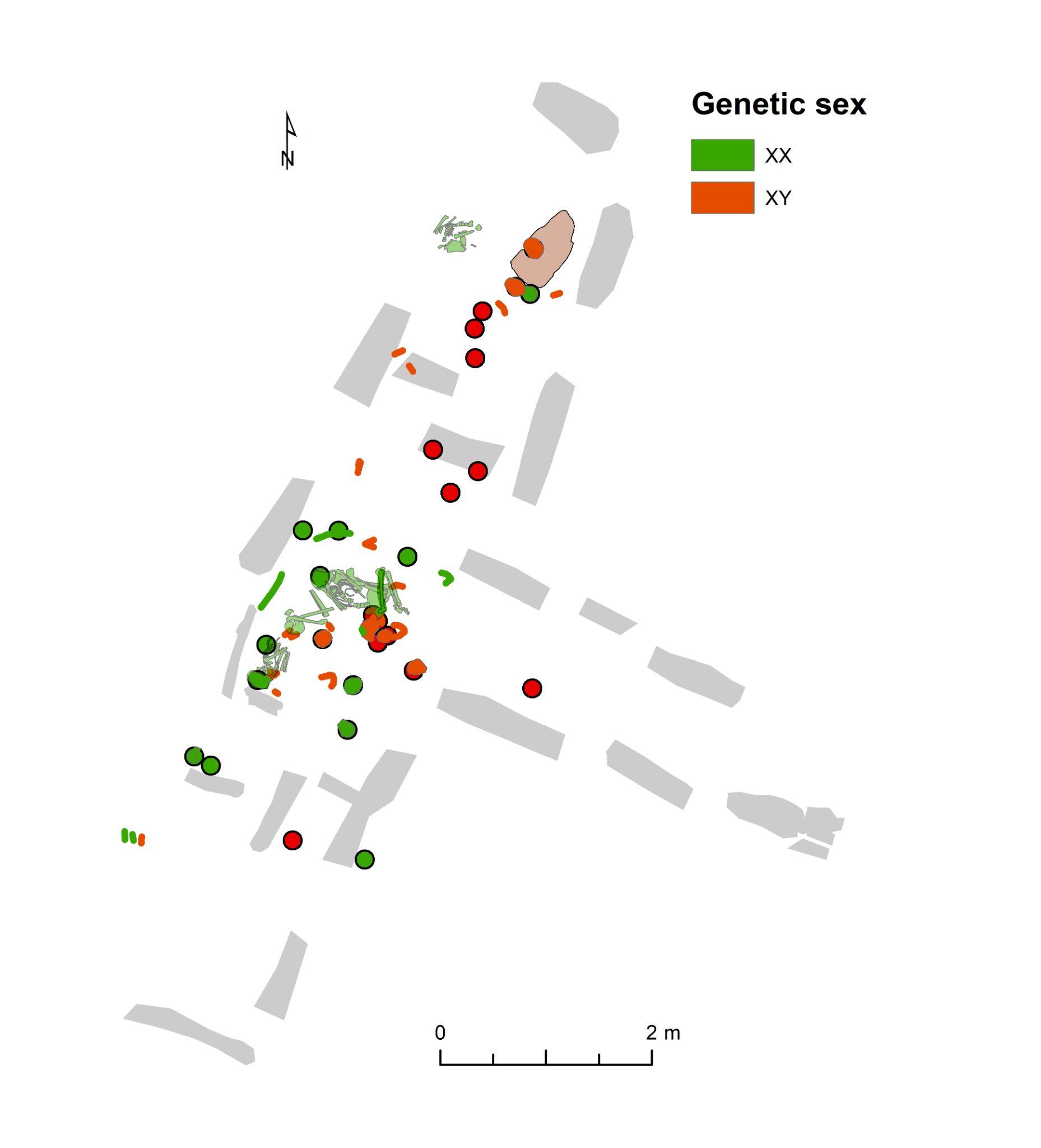
**

**Supplementary Figure 4. Burial locations within the Frälsegården passage grave coloured by sex.**


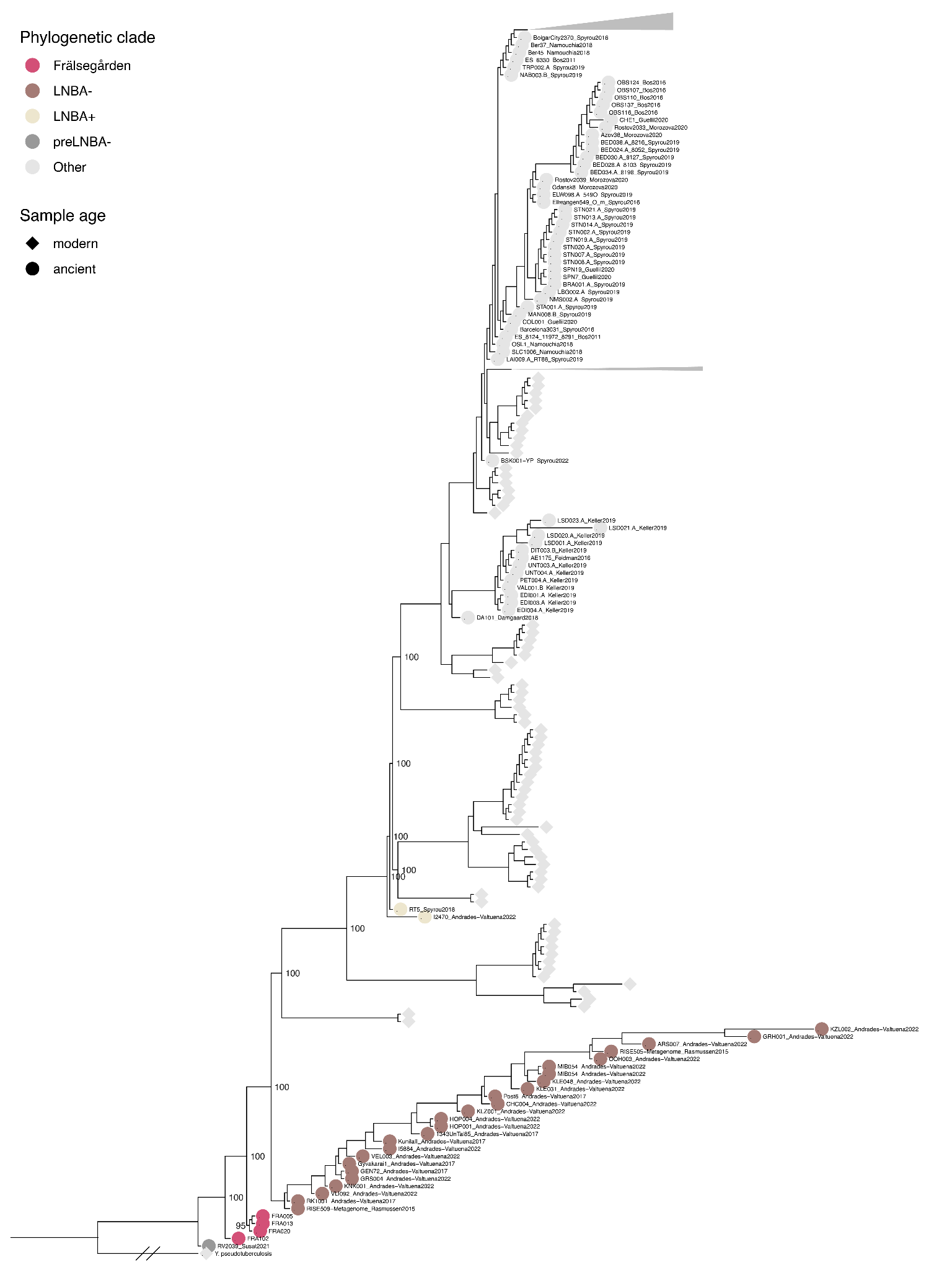


**Supplementary Figure 5. Full phylogenetic tree.** Phylogenetic relationship between all previously published plague strains and the data produced for this study. Each circle represents one plague genome. Phylogenetic clades relevant for this study have been highlighted in colour and only ancient samples were labelled. Transfer Bootstrap Expectation (TBE) values are shown for relevant nodes.


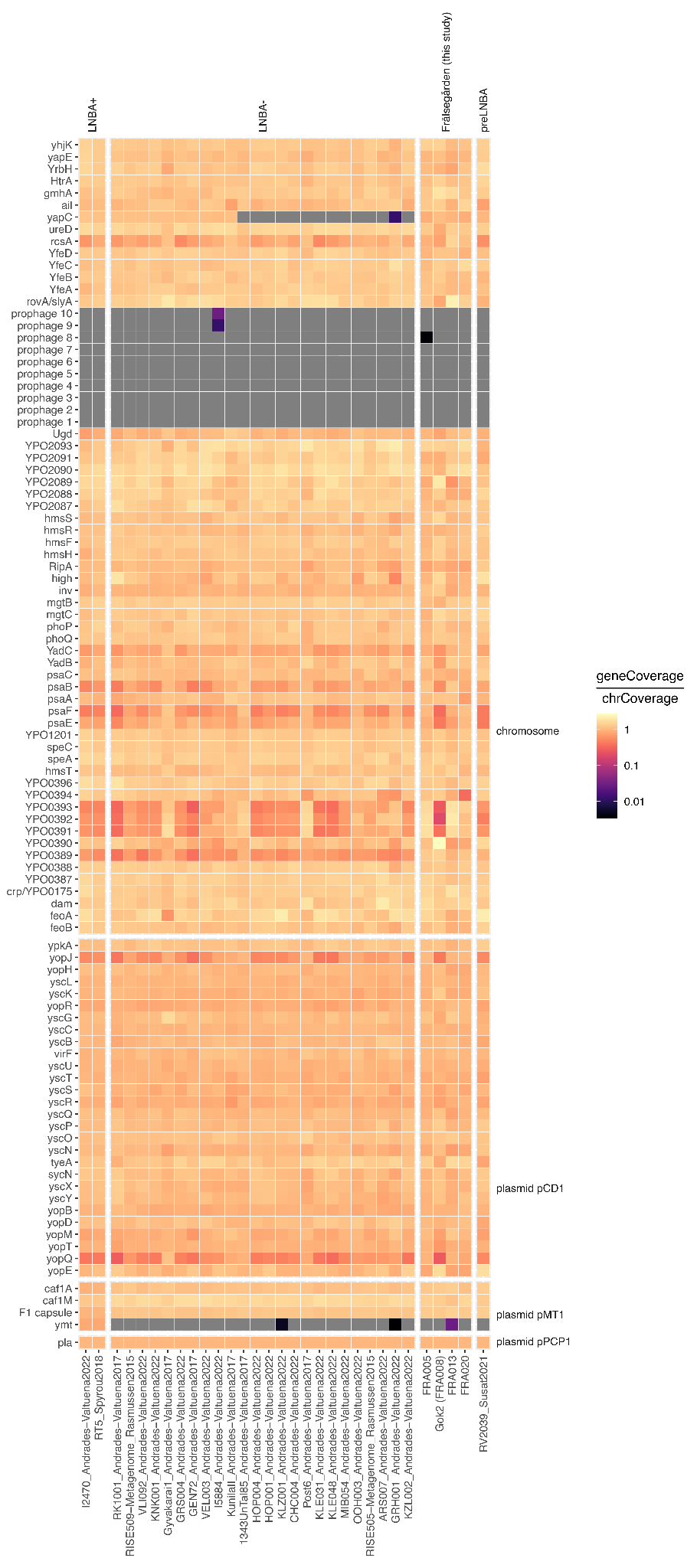


**Supplementary Figure 6. Coverage of known virulence factors.** The four higher-coverage shotgun strains from Frälsegården were included, as well as ancient reference strains from the clades LNBA+, LNBA- and preLNBA. Each gene is coloured by the deviation from the mean coverage, log transformed.


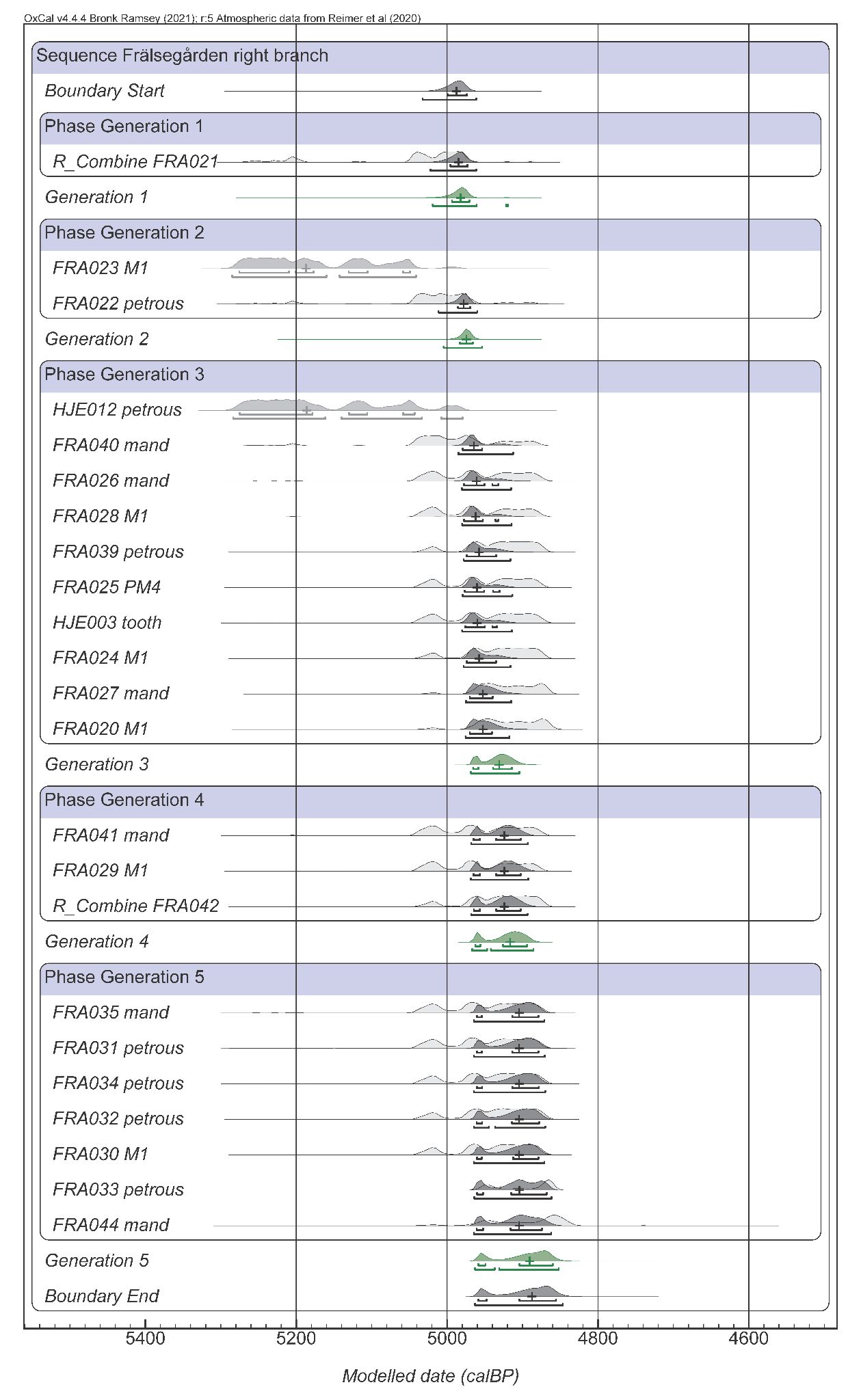


**Supplementary Figure 7. Oxcal plot of modelled dates, right side branch at Frälsegården.** Median dates and 2sd ranges are indicated. Two unmodelled outlier dates are shown in grey (FRA023, HJE012).


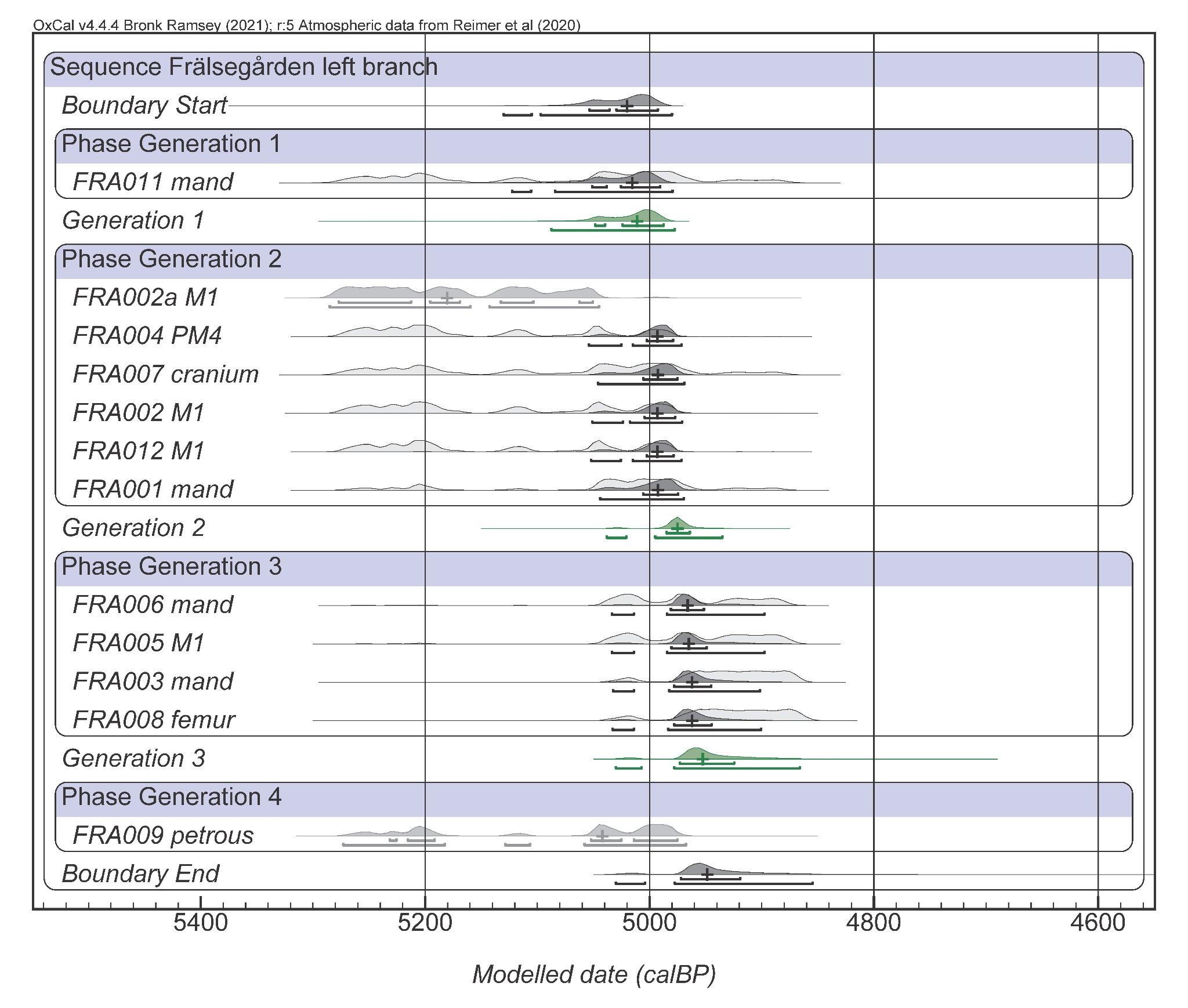


**Supplementary Figure 8. Oxcal plot of modelled dates, left side branch at Frälsegården.** Median dates and 2sd ranges are indicated. Two unmodelled outlier dates are shown in grey (FRA002a, FRA009).


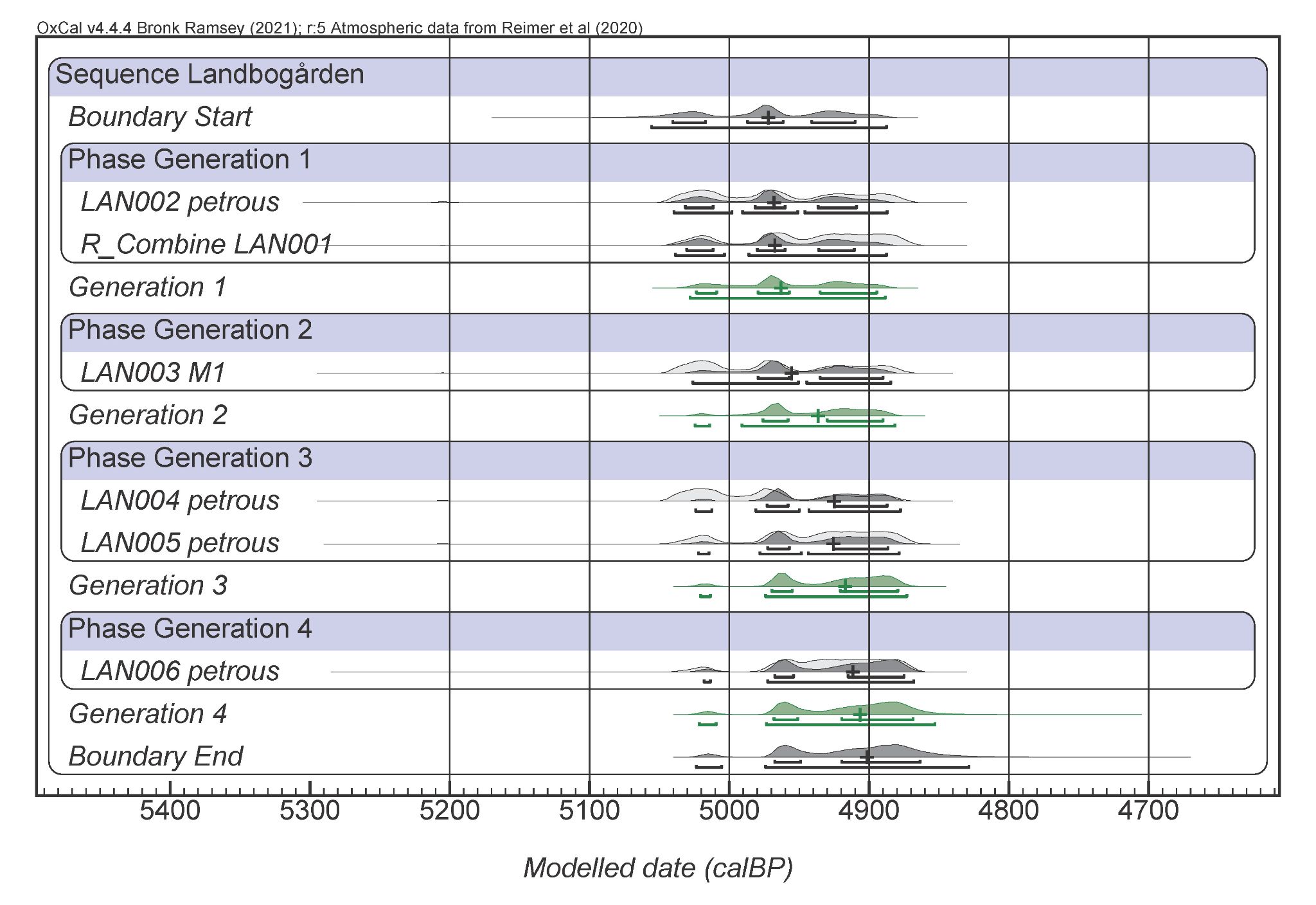


**Supplementary Figure 9. Oxcal plot of modelled dates, Landbogården.** Median dates and 2sd ranges are indicated.

##
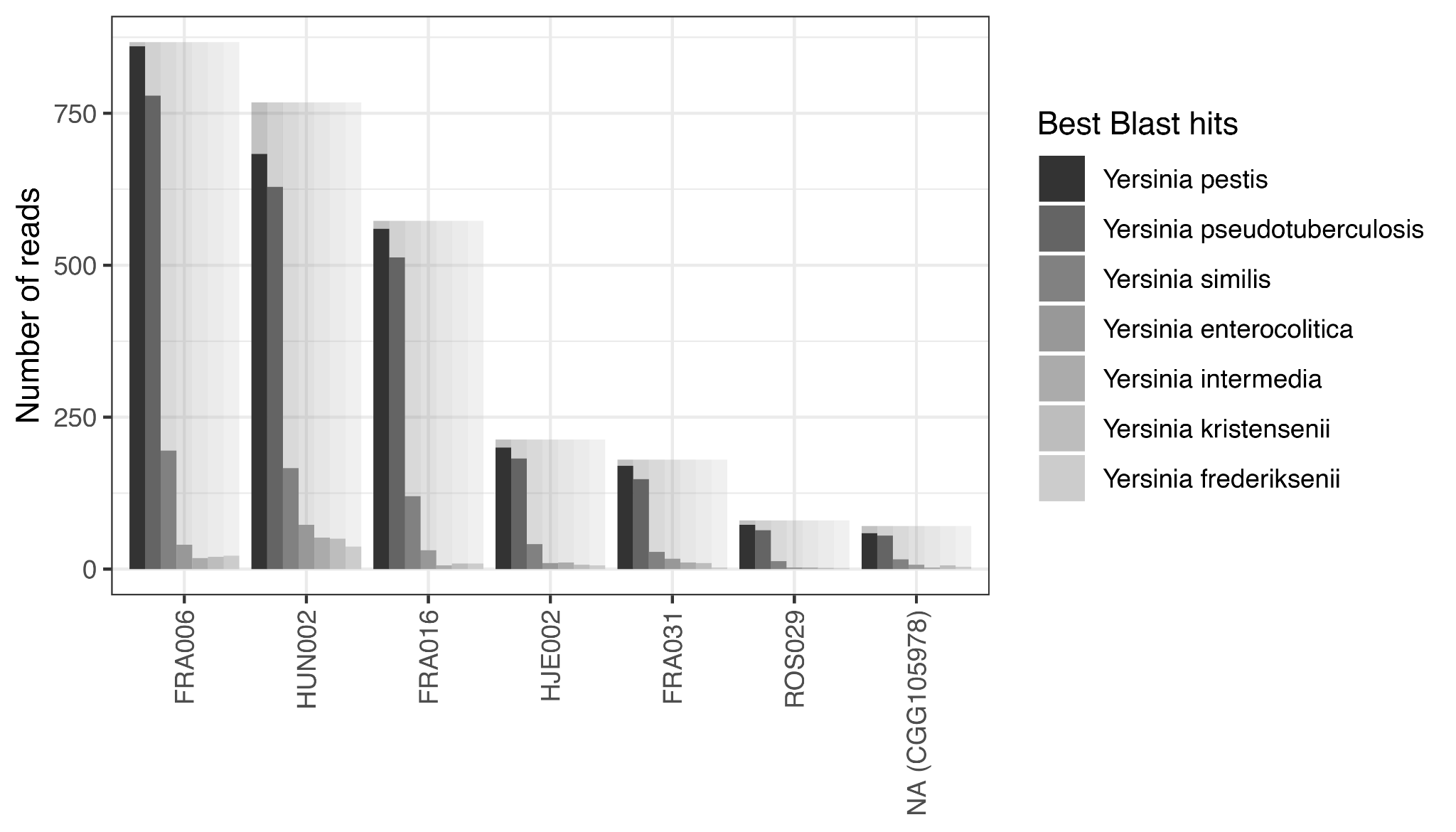


**Supplementary Figure 10. Blast results for tentative plague detections (<0.01X).** Number of reads where a given species was among the best blast hits as defined by the Blast eValue. Shaded bars represent the total number of input reads. Only the seven most common taxa are shown.


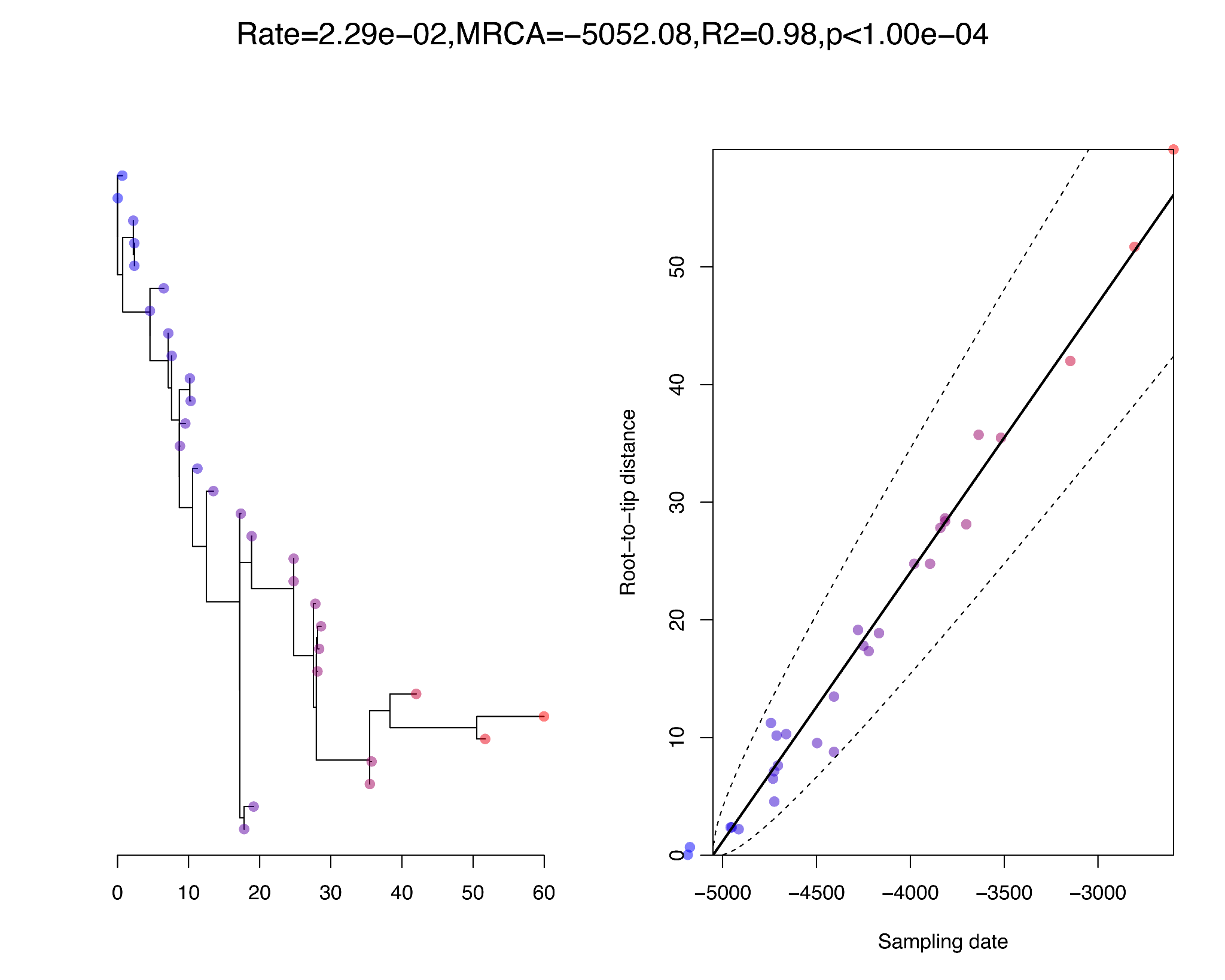


**Supplementary Figure 11. Temporal signal assessment.** Root-to-tip analysis carried out using BactDating.

## Supplementary References

(continued from main text)

74. Iversen, R. *The Transformation of Neolithic Societies: An Eastern Danish Perspective on the 3rd Millennium BC*. (2015).

75. Becker. *Den Grubekeramiske Kultur i Danmark*. (1951).

76. Jankavs, P. Vem var Firse? / Who was Firse? in *Med hjärta och hjärna. En vänbok till professor Elisabeth Arwill-Nordblad* (2014).

77. Blank, M. Bronze Age burials in megalithic graves in Falbygden. in *New Perspectives on the Bronze Age* (eds. Bergerbrant, S. & Wessman, A.) 19–36 (Archaeopress, 2017). doi:10.2307/j.ctv1pzk2c1.7.

78. Blank, M. *et al.* Mobility patterns in inland southwestern Sweden during the Neolithic and Early Bronze Age. *Archaeol. Anthropol. Sci.* **13**, (2021).

79. Skoglund, P. Genomic Diversity and Admixture Differs for Stone-Age Scandinavian Foragers and Farmers. *Science* (2014) doi:10.1126/science.356262.

80. Sjögren, K.-G. Modeling middle Neolithic funnel beaker diet on Falbygden, Sweden. *J. Archaeol. Sci. Rep.* **12**, 295–306 (2017).

81. Skoglund, P. *et al.* Origins and genetic legacy of Neolithic farmers and hunter-gatherers in Europe. *Science* **336**, 466–469 (2012).

82. Malmström, H. *et al.* Ancient DNA Reveals Lack of Continuity between Neolithic Hunter-Gatherers and Contemporary Scandinavians. *Curr. Biol.* **19**, 1758–1762 (2009).

83. Malmström, H. *et al.* Ancient mitochondrial DNA from the northern fringe of the Neolithic farming expansion in Europe sheds light on the dispersion process. *Philos. Trans. R. Soc. B Biol. Sci.* **370**, 20130373 (2015).

84. Persson, P. & Sjögren, K.-G. *Falbygdens Gånggrifter. Del 1. Undersökningar 1985-1998*. (Göteborg University, Göteborg, 2001).

85. Strinnholm, A. Falköping stad 3: Arkeologisk undersökning av en gånggrift. (1996).

86. Axelsson, T. & Persson, P. *Rapport. Undersökning Av Gånggrift. Raä Nr 3, Falköpings Stad, Västergötland.* (1995).

87. Axelsson, T. & Persson, P. *Rapport. Arkeologisk Undersökning 1998, Gånggriften Hjelmars Rör, Raä Nr 3, Falköping Stad, Västergötland.* (1999).

88. Wilhelmson, H. *Hjelmars Rör, En Jämförande Studie Av Ett Återuppgrävt Gånggriftsmaterial Från Falbygden.* (2003).

89. Bägerfeldt, L. *Västergötland Gökhems Socken Landbogården 11:1 RAÄ 17 Undersökning Av En Gånggrift 1987*. (1987).

90. Liden, K., Takahashi, C. & Nelson, D. E. The Effects of Lipids in Stable Carbon Isotope Analysis and the Effects of NaOH Treatment on the Composition of Extracted Bone Collagen. *J. Archaeol. Sci.* **22**, 321–326 (1995).

91. Bägerfeldt, L. *Megalitgravarna i Sverige, Typ, Tid, Rum Och Social Miljö. 2: A Reviderade Upplagan*. (1992).

92. Sjögren, K.-G. C-14 CHRONOLOGY OF SCANDINAVIAN MEGALITHIC TOMBS. in *Exploring time and matter in prehistoric monuments: absolute chronology and rare rocks in European megaliths: proceedings of the 2nd European Megalithic Studies Group Meeting, (Seville, Spain, november 2008)* (2011).

93. Cullberg, C. *Megalitgraven i Rössberga*. 126 (1963).

94. Hedges, R. E. M. & Sykes, B. Biomolecular archaeology: past, present and future. *Proceedings of the British Academy* **77**, 267–283 (1992).

95. Linderholm, A. Migration in Prehistory : DNA and stable isotope analyses of Swedish skeletal material. (Stockholm University, Faculty of Humanities, Department of Archaeology and Classical Studies.m, 2008).

96. Malmström, H. *et al.* The genomic ancestry of the Scandinavian Battle Axe Culture people and their relation to the broader Corded Ware horizon. *Proc. R. Soc. B Biol. Sci.* **286**, (2019).

97. Henriksen, R. A., Zhao, L. & Korneliussen, T. S. NGSNGS: next-generation simulator for next-generation sequencing data. *Bioinformatics* **39**, btad041 (2023).

98. Patterson, N., Price, A. L. & Reich, D. Population Structure and Eigenanalysis. *PLOS Genet.* **2**, e190 (2006).

99. Al, P. *et al.* Principal components analysis corrects for stratification in genome-wide association studies. *Nat. Genet.* **38**, (2006).

100. Maples, B. K., Gravel, S., Kenny, E. E. & Bustamante, C. D. RFMix: A Discriminative Modeling Approach for Rapid and Robust Local-Ancestry Inference. *Am. J. Hum. Genet.* **93**, 278 (2013).

101. Bouckaert, R. *et al.* BEAST 2.5: An advanced software platform for Bayesian evolutionary analysis. *PLOS Comput. Biol.* **15**, e1006650 (2019).

102. Didelot, X., Croucher, N. J., Bentley, S. D., Harris, S. R. & Wilson, D. J. Bayesian inference of ancestral dates on bacterial phylogenetic trees. *Nucleic Acids Res.* **46**, e134 (2018).

103. Coutinho, A. *et al.* The Neolithic Pitted Ware culture foragers were culturally but not genetically influenced by the Battle Axe culture herders. *Am. J. Phys. Anthropol.* **172**, 638–649 (2020).
