# Supplementary material for: Repeated plague infections across six generations of Neolithic Farmers
Source: Nature. 2024 Jul 10;632(8023):114–21. doi: 10.1038/s41586-024-07651-2 (PMC11291285; doi:10.1038/s41586-024-07651-2)
Supplement: Supplementary file 2 — Reporting Summary [file 41586_2024_7651_MOESM2_ESM.pdf]

nature portfolio

Corresponding author(s): Frederik Seersholm & Martin Sikora

Last updated by author(s): May 8, 2024

Reporting Summary

Nature Portfolio wishes to improve the reproducibility of the work that we publish. This form provides structure for consistency and transparency in reporting. For further information on Nature Portfolio policies, see our Editorial Policies and the Editorial Policy Checklist.

Statistics

For all statistical analyses, confirm that the following items are present in the figure legend, table legend, main text, or Methods section.

n/a	Confirmed	
☐	☒	The exact sample size (*n*) for each experimental group/condition, given as a discrete number and unit of measurement
☐	☒	A statement on whether measurements were taken from distinct samples or whether the same sample was measured repeatedly
☒	☐	The statistical test(s) used AND whether they are one- or two-sided<br>*Only common tests should be described solely by name; describe more complex techniques in the Methods section.*
☒	☐	A description of all covariates tested
☒	☐	A description of any assumptions or corrections, such as tests of normality and adjustment for multiple comparisons
☐	☒	A full description of the statistical parameters including central tendency (e.g. means) or other basic estimates (e.g. regression coefficient) AND variation (e.g. standard deviation) or associated estimates of uncertainty (e.g. confidence intervals)
☒	☐	For null hypothesis testing, the test statistic (e.g. *F*, *t*, *r*) with confidence intervals, effect sizes, degrees of freedom and *P* value noted<br>*Give P values as exact values whenever suitable.*
☒	☐	For Bayesian analysis, information on the choice of priors and Markov chain Monte Carlo settings
☒	☐	For hierarchical and complex designs, identification of the appropriate level for tests and full reporting of outcomes
☒	☐	Estimates of effect sizes (e.g. Cohen's *d*, Pearson's *r*), indicating how they were calculated

Our web collection on statistics for biologists contains articles on many of the points above.

Software and code

Policy information about availability of computer code

Data collection: Illumina NovaSeq system

Data analysis:

bcftools (1.16)
bedtools (v2.31.0)
convertf (version 5000)
epa-ng (v0.3.8)
gappa (v0.8.0)
GATK (4.3.0.0)
gcta64 (v1.94.1)
GLIMPSE (v1.1.1)
guppy (v1.1.alpha19-0-g807f6f3)
krakenuniq (0.5.7)
mapDamage (2.2.0-86-g81d0aca)
metadmg ()
minimap2 (2.24-r1122)
mosdepth (0.3.3)
openjdk (1.8.0_92)
pathPhynder (1.a)
phynder
picard MarkDuplicates (2.27.4)
plink (v1.90b6.21)

primus (v1.9.0)
raxml-ng (1.2.0)
samtools (1.17)
seqtk (1.3-r106)
smartpca (eigensoft v. 8.0.0)
yhaplo (1.1.2)
KIN (0.1.0)
KINgaroo (0.1.0)

R packages:
viridis_0.6.2
viridisLite_0.4.1
treeio_1.22.0
tidyr_1.3.0
stringr_1.5.0
scales_1.2.1
readr_2.1.4
RColorBrewer_1.1-3
purrr_1.0.1
kinship2_1.9.6
quadprog_1.5-8
Matrix_1.5-4
igraph_1.4.2
googlesheets4_1.1.0
ggtree_3.6.2
ggh4x_0.2.4
ggplot2_3.4.2
forcats_1.0.0
dplyr_1.1.1
doParallel_1.0.17
iterators_1.0.14
foreach_1.5.2

For manuscripts utilizing custom algorithms or software that are central to the research but not yet described in published literature, software must be made available to editors and reviewers. We strongly encourage code deposition in a community repository (e.g. GitHub). See the Nature Portfolio guidelines for submitting code & software for further information.

Data

Policy information about availability of data

All manuscripts must include a data availability statement. This statement should provide the following information, where applicable:

- Accession codes, unique identifiers, or web links for publicly available datasets
- A description of any restrictions on data availability
- For clinical datasets or third party data, please ensure that the statement adheres to our policy

Fastq files will be made available on the European Nucleotide Archive upon publication of the manuscript.

Research involving human participants, their data, or biological material

Policy information about studies with human participants or human data. See also policy information about sex, gender (identity/presentation), and sexual orientation and race, ethnicity and racism.

Reporting on sex and gender	Not appllicable
Reporting on race, ethnicity, or other socially relevant groupings	Not appllicable
Population characteristics	Not appllicable
Recruitment	Not appllicable
Ethics oversight	Not appllicable

Note that full information on the approval of the study protocol must also be provided in the manuscript.

Field-specific reporting

Please select the one below that is the best fit for your research. If you are not sure, read the appropriate sections before making your selection.

☒ Life sciences ☐ Behavioural & social sciences ☐ Ecological, evolutionary & environmental sciences

Life sciences study design

All studies must disclose on these points even when the disclosure is negative.

Sample size	No tests were carried out to predetermine sample size. Sample size was determined by the availability of archaeological material, and on the DNA preservation in these samples.
Data exclusions	7 DNA libraries were excluded from this study based on high contamination estimates as determined by ContamMix (v1.0.10; please see Supplementary Table 3 for details). Furthermore, another 3 libraries were flagged as suspected sample swaps and excluded because they differed fundamentally with other libraries from the same sample. Lastly all samples with a final depth of coverage under 0.01X were excluded from downstream analyses.
Replication	Out of the 109 genetic individuals analysed in this study, 95 individuals are represented by more than one sequencing library. Having multiple sequencing libraries for each sample serves to validate sequencing results and to pinpoint potential sample swaps. Apart from this type of replication, replication of experimental findings is generally not applicable for this kind of ancient DNA study because of the unique nature of ancient human remains.
Randomization	Not appllicable
Blinding	Not appllicable

Reporting for specific materials, systems and methods

We require information from authors about some types of materials, experimental systems and methods used in many studies. Here, indicate whether each material, system or method listed is relevant to your study. If you are not sure if a list item applies to your research, read the appropriate section before selecting a response.

Materials & experimental systems

n/a	Involved in the study	
☒	☐	Antibodies
☒	☐	Eukaryotic cell lines
☐	☒	Palaeontology and archaeology
☒	☐	Animals and other organisms
☒	☐	Clinical data
☒	☐	Dual use research of concern
☒	☐	Plants

Methods

n/a	Involved in the study	
☒	☐	ChIP-seq
☒	☐	Flow cytometry
☒	☐	MRI-based neuroimaging

Palaeontology and Archaeology

Specimen provenance	Hunnebostrand (SHM 7532:107a+b) was sampled in 2006 at Statens Historiska Museum (SHM) in Stockholm by verbal agreement with Leena Drenzel, SHM. Frälsegården (VGM 1M16-107047) is stored in-house at the Department of Historical Studies, Gothenburg university. Sampling for DNA and other analyses is ongoing by verbal agreement with Maria Vretemark, Västergötlands museum, Skara. Avleberg (NM A 37692-701) is stored at the National Museum, Copenhagen. It was sampled and published by a previous project (Allentoft et al 2022, 2023). The samples from Rössberga were analysed as part of the Atlas project in 2013 under permit number 33-696-2013, issued by SHM (Statens Historiska Museum). The tooth from Firse sten was sampled in 2019-10-30 as part of the project Megalitgravar på Falbygden with permission from Västergötlands museum.
Specimen deposition	Leftover DNA digests, extract and sequencing libraries are stored at the DNA laboratory facilities at Globe Institute, Copenhagen. Upon completion of this project, leftover bone material will be returned to respective museum or university collections from which they were sampled (see above).
Dating methods	Datings were performed at the Keck carbon cycle AMS facility, University of California, Irving. The samples were decalcified in 1N HCl, gelatinized at 60°C and pH 2, and ultrafiltered to select a high molecular wt fraction (>30kDa). δ13C and δ15N values were measured to a precision of <0.1‰ and <0.2‰, respectively, on aliquots of ultrafiltered collagen, using a Fisons NA1500NC elemental analyzer/ Finnigan Delta Plus isotope ratio mass spectrometer. Datings were calibrated in Oxcal 4.4.4 using the Intcal20 calibration curve.

☒ Tick this box to confirm that the raw and calibrated dates are available in the paper or in Supplementary Information.

Ethics oversight	No ethical approval was required for this study.

Note that full information on the approval of the study protocol must also be provided in the manuscript.
